# Reflections of Homeless Women and Women with Mental Health Challenges on Breast and Cervical Cancer Screening Decisions: Power, Trust, and Communication with Care Providers

**DOI:** 10.3389/fpubh.2018.00030

**Published:** 2018-02-28

**Authors:** Catherine Claire Moravac

**Affiliations:** ^1^Department of Medical Imaging, Faculty of Medicine, University of Toronto, Toronto, ON, Canada

**Keywords:** homeless, mental health, cancer screening, mammogram, Papanicolaou, qualitative

## Abstract

This study conducted in Toronto, Canada, explored the perceptions of women living in homeless shelters and women with severe mental health challenges about the factors influencing their decision-making processes regarding breast and cervical cancer screening. Twenty-six in-depth qualitative interviews were conducted. The objectives of this research were (i) to provide new insights about women’s decision-making processes, (ii) to describe the barriers to and facilitators for breast and cervical cancer screening, and (iii) to offer recommendations for future outreach, education, and screening initiatives developed specifically for under/never-screened marginalized women living in urban centers. This exploratory study utilized thematic analysis to broaden our understanding about women’s decision-making processes. A constructed ontology^1^ was used in an attempt to understand and describe participants’ constructed realities. The epistemological framework was subjective and reflected co-created knowledge. The approach was hegemonic, values-based, and context-specific. The aim of the analysis was to focus on meanings and actions with a broader view to identify the interplay between participants’ narratives and social structures, medical praxis, and policy implications. Results from 26 qualitative interviews conducted in 2013–2014 provided insights on both positive and negative prior cancer screening experiences, the role of power and trust in women’s decision-making, and areas for improvement in health care provider/patient interactions. Outcomes of this investigation contribute to the future development of appropriately designed intervention programs for marginalized women, as well as for sensitivity training for health care providers. Tailored and effective health promotion strategies leading to life-long cancer screening behaviors among marginalized women may improve clinical outcomes, decrease treatment costs, and save lives.

## What is Known About This Topic

Low socioeconomic status is associated with under/never-screening for breast and cervical cancer. Homeless women and women with severe mental health challenges are among this group.

## What This Paper Adds

This article, the first qualitative investigation on this topic with homeless women, describes the factors associated with women’s decisions about participating in cancer screening. It includes recommendations to increase cancer screening rates among marginalized women living in urban centers.

## Introduction

### National, Provincial, and Local Breast Cancer Screening Rates in Canada

It is estimated that in 2017 there will be 26,300 new cases of breast cancer detected in Ontario this year and that 1,950 Ontario-residing women will die from the disease. Ontario is 1 of the 10 provinces and 3 territories in Canada. The number of predicted new cases and deaths in Ontario is higher than in any other Canadian province (1). Since the advent of breast screening programs across Canada in the late 1980’s, mammogram use has increased significantly. Although breast cancer prevention initiatives have largely been successful, more than one quarter of Canadian women aged 50–69 report that they have not had a mammogram in the previous 2 years (2). Non-use of mammography has been associated with: being an immigrant (in Canada less than 10 years), living in a low-income household, not having a regular physician, and/or smoking (2). Low social economic status has been associated with (i) the belief that having a mammogram is unnecessary and (ii) failure to return for subsequent breast cancer screening (3).

The current guidelines in Ontario, Canada, recommend asymptomatic women participate in mammography every 2 years starting at age 50 for early detection of breast cancer. According to the Canadian Quality Control Index 2017 (4) between 2014 and 2015, 61.3% of Ontario-residing women between the ages of 50–74 participated in mammography, while the rate of participation in the Toronto Central region during this period was 61.5%. This rate is significantly lower than the national breast screening target rate of 70%, clearly underlining the need for enhanced outreach and educational initiatives in this local region.

### National, Provincial and Local Cervical Cancer Screening Rates

Cervical cancer is caused by the human papillomavirus, a common sexually transmitted infection (5). It can be prevented through participation in regular Papanicolaou (Pap) tests (6). The current guidelines for cervical cancer screening in Ontario recommend that screening commence at age 21 among sexually active women and that Pap tests be conducted every 3 years when results are normal. Abnormal Pap test results should be followed up on a case by case basis depending on the classification of abnormality. Cervical cancer screening can cease at the age of 70 if three or more Pap tests conducted in the past 10 years yield normal results (6).

Canadian Cancer Statistics 2017 projects that 1,550 Canadian women will be diagnosed with cervical cancer this year and 400 will die from the disease. Women who have never or seldom been screened make up more than 50% of all new cervical cancer diagnoses in this country (7). The projected number of cervical cancer diagnoses in Ontario for this year is 710, of whom 150 are expected die. According to data published by the Cancer Quality Council of Ontario, between 2013 and 2015, 61% of eligible Canadian women between the ages of 21–69 participated in Pap tests. During this same time period 57% of eligible women living in the Toronto Central region were screened for cervical cancer. This rate is significantly below the provincial screening target of 85% and represents the lowest screening rate of all 14 health regions in Ontario. Therefore, the Toronto Central region was an appropriate geographical region in which to study women’s attitudes toward both mammography and Pap tests.

### Discrepancies in International Literature on Cancer Screening Rates among Homeless Women and Women with Mental Health Challenges

In Canada, it has been shown that women living in the lowest income neighborhoods have substantially lower cervical and breast cancer screening rates than women residing in the highest income neighborhoods (8). International research about screening rates of highly marginalized women such as those living in homeless shelters and supportive mental health care residences is scarce, contradictory, and often difficult to compare due to variation in sample sizes, demographics, and study design (9–28). The majority of these studies are quantitative with screening statistics often based on self report rather than clinical records. Despite this, there is a generally held belief that homeless women, many of whom have mental health challenges, are at high risk for several types of cancers (17, 29, 30) and carry a disproportionate burden of breast and cervical cancer (31–33). Very few qualitative studies however have been conducted on this topic with women experiencing severe mental health challenges (26, 34) and no qualitative studies are known to exist on screening beliefs and experiences of homeless shelter-residing women. This has resulted in a very poor understanding of why some individuals are rarely or never screened, what motivates others to be screened and why some women miss these prevention opportunities altogether. This study aims to: (i) provide new insights from highly marginalized women about their cancer screening decisions, (ii) describe the barriers and facilitators to breast and cervical cancer screening they experience, and (iii) offer recommendations for future outreach, education, and screening initiatives developed specifically for under/never-screened marginalized women living in urban centers.

## Materials and Methods

### Purpose

The primary research question for this investigation was: (i) What factors influence breast and cervical cancer screening decisions among homeless women and women with mental health challenges residing in Toronto, Canada? Secondary research questions were (ii) What are the barriers to and facilitators for breast and cervical cancer screening among this group of women? and (iii) What recommendations can be made for future outreach, education and screening initiatives developed specifically for under/never-screened women living in urban centers at the individual, organizational, community, and systems levels?

### Approach

An exploratory and reflexive study employing one-to-one qualitative interviews was conducted using thematic analysis for a master’s thesis and dissertation (35). A constructed ontology was used in an attempt to understand and describe participants’ constructed realities. This was done with an appreciation for the complexity of the topic and an awareness of my positionality within the research. The epistemological framework was subjective and reflected knowledge that was co-created by the participants and myself. The approach was hegemonic, values-based, and context-specific. Braun and Clarke (36) define thematic analysis as a method for identifying, analyzing, and reporting patterns (themes) within data. They explain that thematic analysis organizes and describes data sets in rich detail which the researcher then uses to interpret various aspects of the research topic. Boratzis (37) defines thematic analysis as more of a tool than a specific method, however, it is a tool which can be employed across many methods. In the context of this study, it was used as a flexible tool within a constructivist paradigm exploring lived experiences, meaning, decision-making, and the influence of the social determinants of health on beliefs and behaviors.

The approach to this enquiry was guided by an ecological model proposed by McLeroy et al. (38) which recognizes that behavior is influenced by five factors: (i) intrapersonal, (ii) interpersonal, (iii) institutional, (iv) community, and (v) public policy. One of the many strengths of this holistic model is its consideration of the impact that individuals and communities can have on public policy as well as how public policy can have impact on individuals and communities in a bidirectional rather than a unidirectional relationship.

### Eligibility, Sampling Strategy, and Recruitment

Women aged 24–74 living in the homeless shelter system or in assisted living residences (due to mental health challenges) in the Central Toronto area who provided informed consent and who spoke English were eligible for recruitment into the study. “Under-screened” is defined as: any individual who has a cervix who has ever been sexually active who is aged 24 or older and who has not been tested for cervical cancer in more than 3 years, and any individual over the age of 52 years who has not had a mammogram in the past 2 years (exception: breast cancer survivors who have undergone double mastectomy). Ethics approval was provided by Women’s College Hospital Research Ethics Board (2012-004B) in May 2013.

This was a substudy of a larger community-based project entitled “Cancer Awareness: Ready for Education and Screening” (CARES) conducted in the Toronto Central region to provide education and supportive accompaniment to screening primarily for newcomer and immigrant women using a peer model (39). Outreach, education and screening appointments were also provided to women living in homeless shelters, women with mental health challenges and sex trade workers. A stratified sampling procedure was used in the present study in an attempt to gain diverse insights from women who had participated in the educational component of the CARES project as well as those who had not and was inclusive of all screening statuses, i.e., up-to-date, under-screened or never-screened as outlined in Table 1.

**Table 1:** Stratified purposeful sampling framework.

Target groups	Declined education through CARES project (or up to date with both screenings)	Attended education and decided not to be screened	Attended education and decided to be screened	Total
Homeless shelter residing women	4	4	4	12
WMHC residing in assisted living residences	4	4	4	12
Total				*N* = 24

*WMHC, women with mental health challenges*.

Recruitment took place at six sites in Toronto approximately 1 month after education sessions through the CARES project had been provided. Recruitment flyers were posted and on-site office hours were arranged for drop-in enquiries. Oral and written consent was given by all participants including the audio-taping of interviews. Interviews took place in private meeting rooms at participating sites. Questions explored individuals’ thoughts about their own health care in general (both physical and mental), current life stressors, knowledge, attitudes, beliefs, and personal experiences with cervical and breast cancer, Pap tests, and mammograms. The role of fear, fatalism, and apathy in decision-making was also examined. Sociocultural, institutional, and familial influences on decision-making were explored along with perceived barriers and facilitators to cancer screening. At the end of the interview a short questionnaire was administered to gather demographic information and screening history. A modest honorarium was provided to thank participants for their time and contributions. All participants were assigned pseudonyms. Field notes were written after each interview and further reflections documented between interviews.

Audiotapes were professionally transcribed. NVivo11 software was used to organize the data. Initial thematic analysis and constant comparison between cases informed subsequent interviews. Outlier cases were carefully examined. Saturation was believed to have been reached at 20 interviews; 26 interviews were ultimately conducted to ensure rigor. Once all transcripts had been reviewed several times, an initial coding strategy was established. Deeper analysis involved resorting and regrouping of themes and subthemes. Third level analysis resulted in three primary constructs.

## Results

Demographic information and screening history of participants are followed by the outcome of the thematic analyses of interview transcripts.

### Demographics and Screening History

Between September 2013 and March 2014, 26 qualitative interviews were conducted in four settings in Toronto. Two sites were homeless shelters designated for women only. Another site was mixed housing for homeless women along with rental units geared to income with on-site professionals available to assist with substance use and mental health issues. The fourth site was a supportive residence for women with severe mental health challenges who had also experienced housing challenges and homelessness. Invitations to participate in interviews a month after delivery of educational sessions at two other sites did not yield any participants. Both of these sites provided housing and support services for women with mental health and substance use issues.

In all, 50% of the participants reported some postsecondary education; 46% reported some high school education, and 4% reported no education. All participants spoke English. Half of the women had given birth to one or more children. All twenty-six women said that they had provincial health insurance coverage. Eighty-eight percent of the participants reported that they had seen a physician or nurse practitioner in the past year. Of importance however was the fact that at the time of the interview 23% did not have a primary care provider, most of whom were actively seeking one and experiencing difficulties in that quest. Five participants reported having male physicians; 14 had female physicians/nurse practitioners, 5 had no primary care provider, and 2 reported more than one provider representing both genders. Information about screening status and overall health status are summarized in Tables 2 and 3. A total of 13 women were age-eligible for breast cancer screening.

**Table 2:** Breast and cervical cancer screening history.

Cervical cancer screening history	*N* = 26	Breast cancer screening history	*N* = 13
Never screened	1 (3.8%)	Never screened	5 (38.4%)
Underscreened	6 (23.0%)	Underscreened	4 (30.7%)
Up-to-date	19 (73.0%)	Up-to-date	4 (30.7%)

Total UNS	7 (26.9%)	Total UNS	9 (69.1%)

*UNS, under/never screened*.

**Table 3:** Self-reported overall health ratings, *N* = 26.

Poor	Fair	Good	Very good	Excellent
2	8	12	3	1

The majority of participants (77%) rated their overall health as being either fair or good. When asked about level of willingness to have Pap tests in the future 14 indicated that they would be “very willing,” seven were “willing,” one was “somewhat willing,” one was “not sure,” and two said they would be willing if they were sexually active. One woman who had undergone a hysterectomy did not require cervical cancer screening. One participant indicated that she would be willing to have a Pap test 1 year after she has been off all her psychiatric medications.

Thirteen women over the age of 50 were asked about their level of willingness to have mammograms in the future. Seven said that they would be “very willing,” three indicated that they would be “willing” and one said that she would be “somewhat willing.” One woman was “not sure”; one woman was “not willing.” One participant indicated that she would be willing to have a mammogram 5 years after she has been off all her psychiatric medications.

Of particular interest is the fact that two women who were under-screened for cervical cancer were very willing to have Pap tests in the future; others stated conditions under which Pap tests would be considered, and no under/never-screened women refused future cervical cancer screening. Among the five women who had never been screened for breast cancer only one woman was not willing to participate in mammography in the future.

With respect to the distribution of participants who attended CARES information sessions versus those who did not, a summary is provided in Table 4. In one homeless shelter, staff recommended offering the information sessions in two parts on separate occasions. This impacted participation rates in educational opportunities.

**Table 4:** Attendance at CARES Information Sessions.

Attended both sessions	Attended one session	Did not attend session
13 (50%)	6 (23.07%)	7 (26.92%)

In all, 73% of participants received some education about breast and cervical cancer and cancer screening through the CARES Project, while 26% did not. Those that did not participate in educational sessions either decided not to attend or had moved into the facility after the education sessions had taken place. This fulfilled one aspect of the stratified sampling framework, which proposed that approximately two-thirds of the sample would have attended information sessions and one third of the sample would not. Three out of seven women who were underscreened for cervical cancer did not participate in information sessions, and three out of nine women who were under/never-screened for breast cancer likewise did not attend information sessions.

### Results of Thematic Analysis: Contextual Factors, Trust, Communication, and Power

#### Contextual Factors: Mental Health and Substance Use

**Diagram 1:**
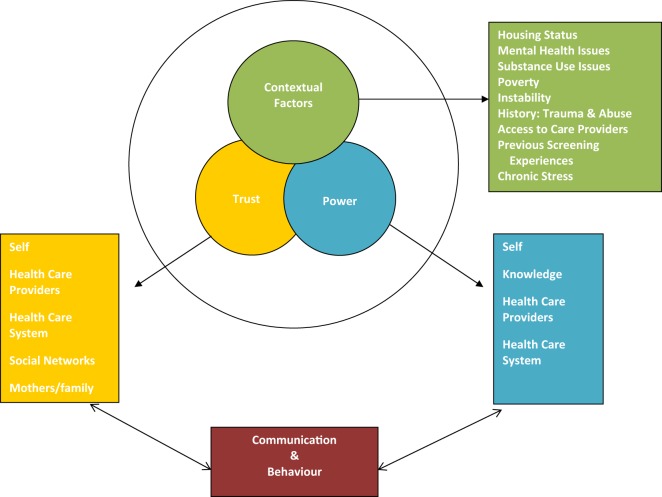
Thematic analysis: factors influencing women’s decisions about cancer screening.

The final analysis of the transcripts brought forth three thematic constructs: contextual factors, trust, and power. Related to trust and power were communication and behavior as shown in Diagram 1. Contextual factors included: mental health, substance use, safe housing, history of sexual abuse, the impact of poverty on maintaining a “healthy lifestyle,” access to primary care, social isolation, and the presence or absence of social networks (35). For some women, mental health challenges did not interfere with participation in cancer screening, while for others, high levels of anxiety for example precluded leaving their residence for any reason. A particularly significant finding was that two women in the study felt that they needed to be “well” before they could be screened for cancer. Andrea, a woman in her mid-50s with serious mental health challenges living in a homeless shelter last had a Pap test 13 years ago; she’d had a mammogram in her 40s due to a family history of breast cancer. She explained that she would probably be willing to have a Pap test and a mammogram in the future but only once she has been off her psychiatric medications for a full year or longer. In her perspective, her body had to be clear of all medications to show accurate results. Another participant, Sophia, a woman in her early 50s with mental health challenges living in a homeless shelter, shared this perception. She explained that her food disorder interfered with regular Pap testing: “*I kind of let that go a bit*.” Like Andrea, she had an idea that she needed to “get normal” or “get healthier” before she would consider going back for this type of check-up:

And my body was going through a whole bunch of different changes through that. And so my logic was with what I’m going through right now any tests that I have now is not going to be completely correct no matter what, because I’m not completely in a healthy status. So my logic was until you get yourself a little better and stronger, when you do, when you feel like you’re somewhere in an average… something you feel that’s back to that normalization then you can go back to doing again your yearly check-ups. Whereas it’s not the best way to look at it but it’s less stressful and less pressure in thinking that right now I have a ton of things I’m worried about. If something is bad I really will not be able to handle that.

Sophia

Women with current or past histories of substance use agreed that their drug use frequently interfered with health care, as Dorothy said:

You know, before, I mean all I thought about is how I’m going to make money and how am I going to buy heroin and just…. No life you know?”…. You just sleep, get up, do your hair and then you go to work to make money [exotic dancing]. That was my life.

Dorothy, a woman in her fifties living in supportive housing for people with mental health and/or addiction issues with previous history of homelessness

She reflected on the fact that when she was addicted she never thought about sickness or about health. In recent years, Dorothy has been taking an active interest in her health, particularly with respect to good nutrition, and has been trying to find a family doctor without success. She had a Pap test 2 years ago at a walk-in clinic, has never had a mammogram but she said she would like to arrange one as soon as she finds a primary care provider willing to accept her as a new patient.

Judy was interviewed at a homeless shelter. She was in her late 20s at the time. When asked what was most important to her about her health she said she was concerned about her mental health, diabetes, physical fitness, and being able to eat healthy foods. Judy divulged that she had been using crystal meth for several years, but had been clean for the past 4 months. She explained that when she was high she would go a week or two without eating. That impacted her blood sugar levels and made her feel unwell. For a period of time, she was in a supportive residence but would not leave her room. She said at that time she didn’t want to talk to anyone and was always depressed. Judy spoke about the impact of her housing situation on her overall health:

Because before I was at another shelter, women’s shelter, called [shelter name]. And … it’s like it’s a nice place. The people are nice. But the activities there (pause) for drug users are very triggering and very addictive.

Judy

During our conversation Judy said what worries her most:

My stability health-wise. And… my housing. Because I know if like this place doesn’t work out and say I end up on the street, I’m pretty much just digging my own grave. You know?

Judy

While addicted Judy did not seek health care, but she reported having had a Pap test a few months ago at the health center on-site and was very willing to keep having them in the future.

#### Contextual Factor: Safe and Stable Housing

Safe housing, not just any housing was reported as being critical to women’s health. Maureen, a woman in her early 40s living in the same homeless shelter as Judy commented on her previous housing situation:

And I’m on anti-depressants. But where I was staying wasn’t very good for me. I was at a shelter before this, it was co-ed and I couldn’t stand it. My depression was really bad. Yeah, I finally got back in here and now I’m much better.

Maureen

Kelly, a woman in her early 60s living in a homeless shelter who had HIV, a life-long history of drug use and other challenges explained that she did have a home but she could not go back to live there. She had been the victim of a violent physical assault in her home, which resulted in a debilitating fear and a high level of anxiety. She spoke about the instability of living within the shelter system:

… there are women here that are here and they become so complacent that you know they’re not even looking you know, but they’re in for a rude awakening because housing and case workers are clamping down and women are being given letters like every day, you know, you got to go, you know, like you got a week. Some get a week, some get a month. I don’t want to leave that way, I want to be able to leave on my own, knowing that I’m going to a place, you know, and I don’t want to be transferred to another shelter. That’s what bothers me the most …

Kelly

#### Contextual Factor: Past Sexual Abuse

A history of sexual abuse is a contextual factor in women’s psychological health and engagement in health promotion activities. The Pap test in particular can often be retraumatizing and lead to avoidance of these cancer checks. Of the 26 women interviewed, 4 disclosed past sexual abuse, 4 others mentioned a history of physical abuse, and 1 woman referenced past trauma.

Nicole, a woman in her early 30s with multiple physical and mental health challenges living at a homeless shelter mentioned that she had been sexually abused as a child. When I asked her about her first Pap test experience and what the health care provider had explained to her, she said that she “*wasn*’*t going to let a man touch me*” and:

So I was glad that it was a female doctor. And she didn’t say oh, it’s in case you get cervical cancer or anything, she didn’t really say why we get one. She just said when you’ve been sexually active. And I didn’t know whether I should include my sexual abuse in that sexually active, because it wasn’t me … you know, participating.

Nicole

Nicole explained that she didn’t know for a long time if she could get a Pap test. She had her first one when she was 25 shortly after she married. Nicole said she didn’t know what to expect, that she found it painful and that she bled afterward. She wasn’t sure if bleeding was normal or not. Despite this she continued to have Pap tests usually every year but had recently lost track of when her next one should be.

Rita was also sexually abused in both her childhood and adult life. Her parents died at a young age; she was in foster care where she was “*continually abused*.” She said that she had delayed having “physicals” done, that she “*wasn’t comfortable with it*.” She admitted to using stall tactics which she ultimately found to be exhausting. When we spoke about her first Pap test she said that it was very painful. She didn’t want to experience Pap tests after that:

I guess it was the bleeding and the uncomfortable of it all. Like I wasn’t-, you know, typically when you go home and you don’t have to worry about any harm coming to you, I was always sexually abused. So I knew if somebody was going to do something or check my body; that it was going to be more painful. So a Pap was more like it’s going to hurt for something else. That’s how I identified.

Rita, a woman in her early fifties living in supportive residence for women with a history of homelessness and/or substance abuse

Despite her feelings, Rita was able to continue to have annual Pap tests during the years when she was raising her children. She said that she recognized the importance of being there for her babies, “*so that kind of clicked in, the responsibility of being that*.” Even though she had found the procedure to be uncomfortable and somewhat traumatic, she was able to tolerate Pap tests for the sake of her children. Later when she started using alcohol and drugs to numb her emotional pain she no longer cared about her health and her attendance and interest in annual physical examinations declined.

Paula, in her late 40s, had a strong aversion to Pap tests. In our conversation, it was implied though not directly stated that her father had sexually abused her. When broaching the subject of Pap tests Paula told me that she was sent to have one when she was a teenager and she didn’t know why or what to expect:

And I didn’t know. And I cried through the whole thing. I just cried. I’ll never forget that. And after that, I thought never again, not a male doctor. [A moment later]: … and he wasn’t (pause) he kept hurting me, and like … I don’t want to talk about it. It was just so—ugh.

Paula

The impact of sexual abuse on cervical cancer screening in Paula’s case was less clear. During the administration of the questionnaire at the end of the interview Paula indicated that she would not be comfortable having another Pap test but she would be willing if it was absolutely necessary. Among the women whom I interviewed who disclosed past sexual abuse I did not find consistent evidence of its negative impact on breast and cervical cancer screening participation. That is not to say that women did not find these procedures difficult. They did however participate in screening. It is not known how many other women in this study may have experienced sexual abuse and been affected differently than the cases discussed.

#### Contextual Factor: Poverty

Living in the context of poverty makes it very difficult to maintain good nutrition, exercise, and a healthy lifestyle as recommended by health care providers, health promoters, and cancer prevention agencies. Several women I interviewed had diabetes; they said it was difficult living in homeless shelters because there weren’t enough healthy sugar-free food substitutions for them at meal times. Frances, a woman in her mid-50s living in a homeless shelter talked about the challenges of being diabetic and not being able to prepare her own food:

Yeah it is [challenging] because I’m supposed to have—diabetes people are supposed to have seven small meals a day. Like breakfast and then a snack, and then lunch, a snack, supper, then a snack. You get the three meals, but you don’t get the snack in between, only after supper.

Frances

Women in poverty who are housed or marginally housed sometimes have to rely on food banks, which in many cases are only able to provide canned goods and non-perishables. It is very difficult to access fresh fruits and vegetables. Simple health promotion messages that fail to attend to these realities can be ineffective and discouraging to marginalized poor women.

### Trust in Health Care Providers and Power Imbalances

Almost a quarter of the women in this study did not have primary care providers. Maureen had been trying to secure a family doctor for a year and a half and had been routinely turned away:

Yeah, especially and a family doctor—I had a hard time finding because I said (pause) I say right up it’s important for me to say I’m an alcoholic. Because I was in recovery for years, but it’s important that they know so that what they prescribe me and all that kind of stuff. Maureen

I was turned away by a few. They said they just can’t deal with even in recovery, and the same thing with the psychiatrist. So that was hard to access.

Maureen

Kelly told me that she was currently looking for a new family physician because she was so “turned off” by the impersonal nature of the intake process, which was conducted by the physician’s nurse:

Yeah to the point where I don’t want to go see the doctor. You know I’ve met her, she’s got my whole history now, I signed the consent forms for all my other health history issues to be sent over to her, like you know my file from [hospital]. But I wouldn’t even call her if I was sick that’s how turned off I was you know.

Kelly

Kelly was disturbed about the fact that she was given a list of tests she would have to undergo before the doctor would see her again. This included among other things, a fecal occult blood test kit and a mammogram:

You know like I saw the paper, the form upstairs for the mammogram. I don’t know when I was supposed to have gone, I think it was within a week of having seen her, you know, but then that day has come and gone and I still have it upstairs and I’m not going. I’m not doing it and I’ll wait and find a doctor that knows how to treat me better.

Kelly

Andrea, a woman in her mid-50s residing in a homeless shelter who identified herself as having been diagnosed with schizophrenia, spoke about Pap tests:

And so I couldn’t do what … You know, worried about pains or … when you can’t even walk, you know. And I can’t even concentrate—watching TV. And you’re telling this doctor that, and she doesn’t give a damn. And why go get a Pap smear or a mammogram when they’d probably ruin you more.

Andrea

Andrea was clear about her decision not to have a Pap test or a mammogram and that decision was embedded in a lack of trust in care providers and a suspicion that the tests could harm her. She added:

I don’t trust doctors. Doctors are not Gods. And they don’t have wisdom. It doesn’t matter if they have years and years of experience. And also, the reason why … five years ago my GP (general practitioner) passed away, and he was a very old man. And he was the best. And you know, he was a gentleman. And he treated me like a lady. And … now I don’t have anybody.

Andrea

Several participants spoke about a lack of trust in physicians. Maureen explained that she had to have Pap tests every 6 months because she had endometriosis. She relayed that the results were always positive so she routinely undergoes biopsies “*just to make sure*.” When asked for more details about the biopsy procedure and how she felt about it she said:

My first one was horrible. It was done by a man who is… it was just the most horrid experience I’d ever had. So it was when they said the next time they wanted to send me for a biopsy I was really skeptical. But it was with my woman gynecologist and it was a much more—I can’t say pleasant. But it was much better handled. She didn’t leave the forceps in me to take a phone call or anything like that. That was horrible.

Maureen

Vanessa, a mature woman who did not disclose her age was living in supportive housing. She was physically well but experiencing mental health challenges following a violent assault. Vanessa shared with me her recollections about poor experiences with both Pap tests and mammograms:

I noticed that in one of the cases there was not a woman with me, the nurse did not come in. So I asked the doctor where was the nurse? And he said things are okay. Don’t worry, don’t worry. So he proceeded with the test. But I decided not to go back to him.

Vanessa

The physician’s behavior resulted in Vanessa’s lack of trust and unwillingness to return. She was eventually able to go on to have Pap tests in spite of this experience. She was resilient and determined to maintain her good health. Unfortunately she continued to have negative experiences:

Because I will ask him, I said could he—because I know that they can warm the instrument a little bit. But he took it from wherever it was. I think it was sterilization. It can be sterilized in heat or in cold I guess. Yeah, he didn’t. It just shocked me. And then with that you know, he didn’t even ask me. I tried to be relaxed. I even talk to myself, relax, relax … But then when the cold came, I just like tightened. Yeah, he kind of told me off. But there have been some good experiences. So I’m not saying that all of them were bad.

Vanessa

Poor interactions with breast imaging technicians also negatively impacted Vanessa’s attitudes toward mammography:

I actually four times [had] experiences that technicians are very rough. And so when the test ended I did go to the reception and let them know that I was not happy with the technicians…. They [said] they would look into it. But I wonder when they’re doing training if that’s something, you know, could be touched upon.

Vanessa

She had different technicians each of the three times that she was treated roughly. In one instance, apart from rudeness, the technician positioned her improperly so that she had to stand on her toes to reach the height of the mammography plate. Vanessa delayed having her fourth mammogram as a result of these disturbing experiences:

The fear of the first three stayed with me. So then I was skipping the appointment because the fear was still there.

Vanessa

Although many interviewees talked about power imbalances, poor communication, and interactions with care providers, being judged, feeling stigmatized, unheard, and rejected, there were also stories about good health providers and trust in their care. Teresa noted that her family doctor wanted her to have a mammogram and even arranged the appointment, but Teresa didn’t follow through. When asked why she said:

Well, I heard they hurt. So I wasn’t all that excited to go. But I’m like this is something I need to do. But then everything else kind of snowballed in my life and that just fell to the backburner as well. … It’s just been me with the follow ups, so just bad timing. But I’m willing to go. That’s one thing; it’s on my list of things I need to do. If she says I should get it done then I trust her.

Teresa

Louisa, an older woman whose physical disabilities require the use of a wheelchair, recounted that her very first mammogram experience was “*Actually quite good. It was exceptional*.” When asked what made the experience exceptional she explained that the technician took the time to find out that it was her first mammogram and spent more time with her, “*compared to today*’*s standards*.” She went on to say that her second and third mammogram experiences were “*not so good*.” Prompted to elaborate she said:

It was let’s get the breasts in there, let’s get it done, let’s get it over, it’s like lunchtime, let’s go. She’s already had her first one, she knows what to expect. Bye-bye. Both times. …. (and later in the conversation). It does mean it gets shoved in the priorities. Because it’s, OK I have to psych myself up for that kind of clinical approach.

Louisa

Louisa also noted that she did not experience pain with her first mammogram but did experience pain during her second and third mammograms when technicians were rushed, inconsiderate, and not very communicative. At the time of the interview she was behind in her breast cancer screening:

I mean if someone doesn’t shove it down my throat and call and say look, can we make this appointment and I’ll meet you at the front door kind of thing, you just don’t bother.

Louisa

### Influence of Other Women on Cancer Screening Decisions

Many of the women interviewed were socially isolated. Only two described receiving support from faith communities; many indicated that they did not have any friends and/or were estranged from family members. Mothers (and grandmothers) however were often mentioned with respect to their personalities, inner strength, past achievements, and also with respect to their influence on screening decisions. In most of these cases, participants’ mothers were no longer alive; they were reflecting on memories. For example, when speaking about cancer screening, Gwen commented:

And that’s my mother. You know, you, this is what you must do, and do it. And so I never was afraid or anything. It’s [screening] important.

Gwen, age 67 living in a supportive residence

Women sometimes spoke about hearing other women say that Pap tests are difficult or mammograms are painful and this sometimes acted as a barrier to screening. Only one example was found in which the influence of others facilitated screening. Frances, a woman in her late-50s who was living in a homeless shelter, said she had never had a mammogram and was quite afraid to have one. However, during the interview she stated that she was going to try to overcome her fear and speak with her doctor about arranging one. When asked how she made this decision, Frances explained that she spoke with other people who said it did not hurt. She said that she was still afraid, but that she should go.

### Participant Recommendations

Some participants were not able to offer specific recommendations about future improvements which could be made to cancer screening programs. The majority of participants had suggestions which have been synthesized below.

Participants’ recommendations for conducting Pap tests

- Ask about sexual history first
- Explain what will happen during the procedure
- Provide reassurance and help to reduce the patient’s fear
- Arrange for a female (nurse or other) to be in attendance
- Enquire if the patient would like the speculum warmed up
- Make suggestions about how the patient can relax
- Be gentle when performing the procedure
- Explain what is happening during the procedure
- After the procedure ask the patient how it went
- After the procedure ask the patient if there is anything that could have been done differently

Other synthesized participant recommendations included:

Participants’ overall recommendations to improve engagement and cancer screening experiences

- Health care providers follow guidelines for conducting Pap tests as outlined above
- Creating less painful procedures to check for breast cancer
- Providing empathy and communication skills training for breast imaging technicians
- Training health care providers to be more respectful, to have more empathy and to provide more time during appointments
- Clerical staff should receive sensitivity and awareness training
- Create volunteer and staff opportunities to provide supportive accompaniment for clients attending medical appointments who need it
- Family physicians should be open to accepting new clients who have complex histories
- Resource materials should be produced in plain language, multiple languages and in large print
- Resource materials should be produced in Braille, on audio cassettes or on DVDs

## Discussion

Study findings suggest that changes need to be made with respect to outreach, education, and cancer screening practices for under/never screened women living in homeless shelters and supportive residences. Adapting public health messaging and education to recognize the contextual factors of women’s lives who have had poor interactions with the health care system and/or who face unique challenges may increase breast and cervical cancer screening among these women. Doing so many decrease health care costs for treating women with cancer that is found at a later stage, decrease the burden of disease among this marginalized group of women, and save lives.

### Cancer Screening Rates

Results of the demographic and screening history questionnaire were encouraging in that only 26.9% of women were under/never-screened for cervical cancer. Given the complex and challenging life circumstances of most of the women interviewed, combined with unpleasant screening experiences by some, this was a surprising outcome. It may reflect the 2012 change in guidelines from annual cervical cancer screening to every 3 years when results are normal. Another reason for these findings may be that on-site Pap tests were available at most sites. The 73% cervical cancer screening participation rate in the past 3 years among the women in this study was primarily higher than the cervical cancer screening uptake rates among homeless women reported elsewhere in the literature (9–12, 14–18).

The mammography screening rate (30.9%) of age-eligible participants in this study was lower than those reported elsewhere in the literature (21–24, 26, 27). Mammograms require women to travel to screening sites and this may explain in part the high rate (69.1%) of under/never screening for mammography among the women in this study. Lower screening rates may also be attributed to the older ages of the women, some of whom may be fatigued by difficulties they’ve experienced interacting with the health care system over the years. Despite this, an encouraging finding was that only one of the never-screened women rejected the idea of future participation in mammography.

Consideration should be given to the use of mobile mammography units so that medically under-served women including women with disabilities, mental health, and housing challenges and women without primary care providers could more easily access screening services in their neighborhoods. Mobile screening programs have been in existence in North America for more than 20 years, although service delivery is usually focused on remote geographical areas (40–48). A cost effectiveness analysis could be conducted to compare operating costs of such a program in a particular urban area with diagnostic and treatment costs for under-served women whose cancer is detected at a late stage.

Despite fear and lack of trust in care providers, almost 77% reported that they were “willing” or “very willing” to have mammograms in the future. A possible explanation for this may be the impact of having attended an information session on the topic, and/or the impact of discussing breast cancer and mammograms during the interview. Another explanation may be that some women who did not have primary care providers intended on having mammograms once they had secured one.

### Barriers and Facilitators to Cancer Screening

Analysis of the interview data revealed the following barriers to cancer screening: fear of the procedure, fear of pain, fear of hearing the results, not having access to a family physician, history of unpleasant/unsatisfactory Pap tests or mammograms, lack of trust in health care providers, other life issues being more highly prioritized, mobility issues, transportation issues, not being well enough psychologically to leave the facility, experiences of discrimination, history of sexual abuse, dis-interested attitudes, and varying levels of knowledge and understanding about cancer checks. Cancer screening decisions among this group of women were varied and complex.

Facilitators to cancer screening included: having access to a family physician; being satisfied with one’s family physician; having had previous Pap tests and/or mammograms that went reasonably well; being encouraged or influenced by mothers, friends, or others in one’s social network; and valuing cancer checks as routine activities that help to maintain good health.

The range of mental health challenges participants faced impeded participation in cancer screening among some and appeared to have no impact on screening decisions or participation among others. Active engagement in substance use negatively impacted screening activities, however, once in a stage of sobriety, uptake of cancer screening did occur. Not having stable, safe housing interfered with some women’s ability to engage in screening; however, on-site Pap tests at shelters facilitated screening among others. A past history of sexual abuse can be a barrier for some women participating in routine cervical cancer screening. Despite this, however, there were examples of women with sexual abuse histories who found undergoing Pap tests to be difficult, yet they were able to participate in regular screening.

Women living in poverty have higher priorities to contend with, which over-shadow thoughts about cancer screening. These may include housing needs, need for particular foods or personal care items, access to government funding programs or other health care needs. Access to a health care provider facilitated participation in breast and cervical cancer screening, while lack of access was a barrier to screening. Social isolation may inhibit involvement in cancer screening, while having access to a social network could influence decision-making in either direction.

Lack of trust in health care providers or in the health care system was presented as a barrier to screening for some women. Other interviewees expressed distrust of some health care providers; however, they were able to persist with their commitment to cancer screening by changing physicians. Fear of screening was overcome through trust in care providers; trust in the notion that “it’s for my own good” and self-trust that one could handle it. Communication, both poor and excellent, influenced women’s sense of trust in care providers and in their perceptions about physicians’ greater position of power relative to their own. This data illustrates the fluidity of decision-making and the ways in which power, trust, and complex contextual factors impact on women’s screening decisions. One’s perception about power is influenced by trust. Trust is cultivated in oneself, in health care providers, in health care systems and in social networks.

Many interviewees seemed to think about cancer as you either have it or you do not; there were no discussions about types or stages of cancer. The key health promotion message that finding cancer early offers a better chance of a good outcome than when cancer is found at a later stage was understood by approximately half of the interviewees. Among those for whom the messaging resonated, they did not speak about different degrees of severity of cancer. Some behavior change models assume that: people have ready access to cancer screening information, levels of health literacy are sufficient to process available information, and health and wellness are universally defined, understood, and valued. This was not necessarily the case among women interviewed in this study.

There is a substantial body of extant literature on patient/physician communication, the impact of communication on issues of patient trust, and approaches for providing patient/woman-centered care (49–52). Despite this, there has been growing concern in recent years about the level of patient dissatisfaction with provider communication. Levinson and Pizzo (49) addressed this issue in a commentary published in the *Journal of American Medicine* in which they stated that medical schools and residency programs focus the majority of time on science and technology and minimal time on skill development for effective communication. They also commented on the impact of some physician incentive systems which reward efficiency and high patient volumes resulting in brief appointments and sometimes dissatisfied patients. Recommendations included: allocating more time for patient-centered communication skills in medical schools and residency programs, providing incentive-based professional learning opportunities for practising physicians, regularly implementing patient satisfaction surveys, and conducting annual physician evaluations using 360° models incorporating peer and patient feedback.

Establishing or maintaining patient trust in health care providers is a complex undertaking. A 2013 Cochrane review including 10 randomized control trials did not produce sufficient evidence that any of the interventions impacted on an increase or decrease in patient trust in physicians (52). Several studies have been conducted on physician attitudes toward homeless and low income individuals (53–55) as well as attitudes of drug users toward physicians (56–60). Many of these studies highlight a perception by drug users that they are disrespected and routinely experience prejudice. Lack of physician knowledge or interest in addictions accounted for some of these difficulties.

Primary care providers through their power and control to accept or deny new patients both directly and indirectly affect marginalized women’s access to cancer screening. Unfortunately many Canadian women are unaware of the fact that they can self-refer for routine mammography after the age of 50. A qualitative study conducted in British Columbia, Canada (61) with un-attached marginalized patients with one or more chronic illnesses found that participants worried about lost opportunities for preventive care, not having a consistent medical record, being excluded from care because they were perceived by physicians as being “difficult” cases, and their inability to access referrals to specialists. Focus group participants believed in the benefits of having a family physician and wanted to have the opportunity to develop trusting relationships with care providers. Participants using walk-in clinics reported being treated by different providers each visit, with the result that physicians did not have the continuity of contact to properly assess health care deterioration over time. To partially address these difficulties an effort could be made by health care providers working in walk-in clinics to review preventive care needs after dealing with the primary reason for the visit.

### Women with Mental Health Challenges

A significant finding in this study was the concept that some women with mental health challenges believe that they cannot participate in cancer screening until they feel that they are well, healthy, or normal. This has implications for future practice. Care providers should consider taking the time to listen to women’s rationalizations about cancer screening decisions and help them to better understand how their bodies work. Listening and really hearing how women think about these issues and make decisions may facilitate a different kind of conversation. Such a conversation may also provide greater insight into other aspects of women’s health decision-making.

### Addressing Systemic Barriers

An important outcome of this study for health promotion specialists, health care providers, professional governing bodies, cancer agencies, and policy makers is the need to move away from assumptions about individual-based deficiencies in under-screened women to acknowledging that poor physician/patient encounters are partially responsible for some women’s discontinuation of cancer screening activities. It is frequently presumed that women are deficient in knowledge, awareness, understanding, or the resources needed to attend screening activities. Public messaging, health promotion activities, and educational events assume that once women have the information they will follow through with cancer screening (62–66). Poor interactions with health care providers are not discussed nor is the fact that some women are not able to secure primary care providers to attend to their health care needs in the first place.

For women who have had poor screening experiences, a different approach is needed. Experiencing pain, rudeness, or disrespect cultivates distrust in health care providers and the health care system as a whole, which has larger implications for general health care needs. There is work to be done with respect to sensitivity training, communication skills, and technical skills for professionals. This includes receptionists, administrative assistants, breast imaging technicians, family physicians, gynecologists, oncologists, and other specialists involved in cancer treatment. Increased rates of cancer screening among under/never-screened women will not be achieved until inadequacies in the health care system are addressed in conjunction with improved health promotion strategies.

### Study Limitations

The fact that the analysis of qualitative data was conducted by one researcher rather than a team may be considered a limitation of the study. The postgraduate thesis this manuscript is based on however was supervised by three faculty members who thoroughly critiqued the methods, analysis, and conclusions. Some may question whether the responses to queries about willingness to participate in future screening may have been affected by a wish to please the interviewer, as in the Hawthorne effect. Based on the conversations I had with women, and how I situated myself within those discourses, I do not believe this was the case. Participants were extremely forthcoming about their attitudes and experiences and did not appear to hold back in any way on their opinions.

### Recommendations

**Diagram 2:**
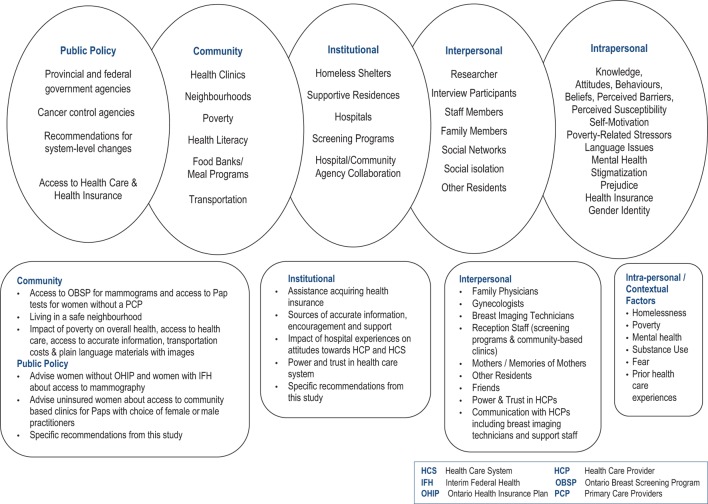
Application of McLeroy et al.’s ecological model to study findings (38).

McElroy’s ecological model provided the framework both for the interviews and the analysis of results. The model emphasizes the complexity of issues underpinning the lack of engagement of marginalized women in cancer screening programs. It also provided a lens through which to propose changes to improve the situation (Diagram 2) at the individual, organizational, community, and public policy levels.

### Individual Level

The individual level of this model includes both intrapersonal and interpersonal factors. Initiatives at the community level, for example public education must take into consideration intrapersonal and contextual factors to be effective. All five domains of this model are interconnected and interdependent and should be conceived of as components of a holistic framework. In the most basic sense, political will and funding at the systems level allows communities and organizations to function in ways that directly impact individuals.

The timing of when a woman is invited to consider cancer screening can play a significant role in her decision-making relative to current life circumstances, level of stress, and competing priorities. The decision-making process is also different for women who have already experienced the procedure versus those who have not, and the quality of the interaction which occurred for those who were screened. Poor experiences are often recounted to other women which in turn impacts those women’s decision-making processes. Health professionals conducting Pap tests should ask women at the outset if there is anything they can do to make women more comfortable. This may allow for an opportunity for women to disclose prior sexual abuse or prior difficulties experienced during cervical cancer screening. Appropriate accommodations can then be made. A strategy should be developed to encourage physicians and nurse practitioners employed at walk-in health clinics to enquire about preventative health care needs during routine appointments for other presenting issues.

One of the most salient outcomes from the present study is the need for health care professionals to interact with women in a respectful, professional, and sensitive manner. Many physicians already practice in this way, as much as they can under existing time constraints. Others use power and control when interacting with patients, negatively impacting on women’s health-based decisions and subsequent access to care. Some health care needs may be left unmet as a result of some women’s normalization of traumatic experiences and reluctance or discomfort to engage in a dialogue about the impacts of those experiences on their health and well-being. The cultivation of attentive, patient, empathic and interpretive listening skills among health care providers will begin to improve this situation. A sensitivity training and communication skills course could be made available to all health care providers along with an incentive for educational credits.

### Organizational Level

The organizational level refers to institutions such as homeless shelters, supportive residences for WMHC, hospitals, and cancer screening programs. Homeless shelters should be provided with adequate funding to periodically offer educational sessions on breast, cervical, and colorectal cancer specifically tailored to the contextual factors that women are dealing with while residing there. These sessions should expand on standard cancer awareness programs to acknowledge poor interactions which sometimes happen with care providers, prejudice and stigmatization, issues of power and trust, trauma, and effective communication strategies when interacting with health care professionals. Suggestions could be made for women who have had negative Pap test experiences to consider: going to a public health unit, to a female provider, or to a community-based clinic and to bring a friend or a person whom they trust.

Women who have had negative mammography experiences could be encouraged to go to a different screening site, to discuss with the technician beforehand what issues they experienced previously, and to ask the technician for specific accommodation. Messaging about early detection of cancer should explain in clear language the continuum in which cancer grows and indicate that different outcomes can occur depending on when women participate in cancer screening should they happen to have cancer. Helping women to understand that having breast cancer does not necessarily mean removal of the entire breast can help reduce fear. Women may also be reassured to know that cervical cancer is preventable when pre-cancerous lesions are found. Thoughtful use of imagery is particularly important when sharing information with women of varying levels of literacy. Photos and images should be relatable to the women participating so that they personally identify with the relevance of the information to their own lives.

Ideally this education would be offered as a component of a series of discussions on topics pertaining to women’s health and well-being. These sessions could be co-facilitated by women who have experienced homelessness, substance use or mental health issues who are interested and stable enough to participate in training to build capacity to fulfill this role. Efforts could be made to engage volunteers and/or staff to coordinate appointments and provide supportive accompaniment to group appointments for cancer screening. Modest success with this model to engage marginalized women in mammography has been reported by Heyding et al. (13) and others (22, 67–69).

Hospitals should consider offering sensitivity training to health care providers, support staff, and breast imaging technicians. An effective format for these educational tools would be the use of film. Several short vignettes featuring women talking about their lives, health conditions, thoughts, and feelings before going for a Pap test or a mammogram, could be followed by an enactment of both a poor provider/patient interaction and an ideal provider/patient interaction.

### Community Level

Cancer awareness educational programs as described above should also be offered in community settings such as drop in centers and special programs for street-involved women, WMHC, substance use issues and other women who experience marginalization. Information sessions tailored to contextual factors in their lives will likely have greater impact and success than information sessions which have been designed for the general public. As in institutional settings, efforts should be made to provide assistance with scheduling appointments and supportive accompaniment to screening. Individuals in this support role should understand and expect that some appointments may be cancelled, however consistent follow-up should be encouraged. Group scheduling of appointments is an efficient approach to use however it may not always be feasible. Adding a pleasant activity to the group scheduling may be helpful. Utilization of a mobile health unit could be considered to visit homeless shelters, residences for WMHC, residences for women with physical disabilities, programs for women with developmental disorders and other places where women are who may find it difficult or impossible to attend screening sites. The bus could be outfitted with adjustable equipment for women who use wheelchairs.

### Systems Level/Public Policy

Permanent ongoing funding for implementation of community based and institution based cancer awareness education programs for marginalized women should be advocated for and obtained. Funding should be sought for the production of sensitivity training tools for health care providers, administrative staff and volunteers. National funding bodies could provide financial support to ensure wide-spread distribution of resource tools.

## Conclusion

Improvements to cancer screening systems currently in place are needed so that homeless women and women with mental health challenges receive accurate and relatable information tailored to the contextual factors of their lives. Health care providers need to be sensitive to women’s lived experiences, and provide patient-centered, empathic care free from discrimination. All women deserve to be treated with respect and dignity.

## Ethics Statement

This study was carried out in accordance with the recommendations of Good Clinical Practice (GCP), The Tri-Council Policy Statement 2 (TCPS2), and the Responsible Conduct for Research (RCR). It was approved by the Research Ethics Board at Women’s College Hospital in Toronto, Ontario, Canada. All participants provided informed, written consent to be interviewed, and audio-taped for this study in accordance with the Declaration of Helsinki.

## Author Contributions

Study conception, design, implementation, data analysis, and manuscript preparation (CM).

## Conflict of Interest Statement

The author declares that the research was conducted in the absence of any commercial or financial relationships that could be construed as a potential conflict of interest.
